# Machine learning-based risk models for procedural complications of radiofrequency ablation for atrial fibrillation

**DOI:** 10.1186/s12911-023-02347-5

**Published:** 2023-11-10

**Authors:** Rong Li, Lan Shen, Wenyan Ma, Linfeng Li, Bo Yan, Yuna Wei, Yao Wang, Changqing Pan, Junyi Yuan

**Affiliations:** 1 Clinical Research Center, Shanghai Chest Hospital, Shanghai Jiao Tong University, Shanghai, China; 2 Yidu Cloud Technology Inc, Beijing, China; 3 Hospital’s Office, Shanghai Chest Hospital, Shanghai Jiao Tong University, Shanghai, China; 4 Information Center, Shanghai Chest Hospital, Shanghai Jiao Tong University, Shanghai, China

**Keywords:** Atrial fibrillation, Radiofrequency ablation, Machine learning, Predictive model

## Abstract

**Background:**

Radiofrequency ablation (RFA) for atrial fibrillation (AF) is associated with a risk of complications. This study aimed to develop and validate risk models for predicting complications after radiofrequency ablation of atrial fibrillation patients.

**Methods:**

This retrospective cohort study included 3365 procedures on 3187 patients with atrial fibrillation at a single medical center from 2018 to 2021. The outcome was the occurrence of postoperative procedural complications during hospitalization. Logistic regression, decision tree, random forest, gradient boosting machine, and extreme gradient boosting were used to develop risk models for any postoperative complications, cardiac effusion/tamponade, and hemorrhage, respectively. Patients’ demographic characteristics, medical history, signs, symptoms at presentation, electrocardiographic features, procedural characteristics, laboratory values, and postoperative complications were collected from the medical record. The prediction results were evaluated by performance metrics (i.e., the area under the receiver operating characteristic curve (AUC), accuracy, sensitivity, specificity, F score, and Brier score) with repeated fivefold cross-validation.

**Results:**

Of the 3365 RFA procedures, there were 62 procedural complications with a rate of 1.84% in the entire cohort. The most common complications were cardiac effusion/tamponade (28 cases, 0.83%), and hemorrhage (21 cases, 0.80%). There was no procedure-related mortality. The machine learning algorithms of random forest (RF) outperformed other models for any complication (AUC 0.721 vs 0.627 to 0.707), and hemorrhage (AUC 0.839 vs 0.649 to 0.794). The extreme gradient boosting (XGBoost) model outperformed other models for cardiac effusion/tamponade (AUC 0.696 vs 0.606 to 0.662).

**Conclusions:**

The developed risk models using machine learning algorithms showed good performance in predicting complications after RFA of AF patients. These models help identify patients at high risk of complications and guiding clinical decision-making.

**Supplementary Information:**

The online version contains supplementary material available at 10.1186/s12911-023-02347-5.

## Background

Atrial fibrillation (AF) is one of the most common sustained heart rhythm disorders and a condition associated with high mortality and morbidity. There are nearly 335 million patients with AF worldwide [1], with a prevalence rate of 2.9% [2]. The incidence of AF is increasing rapidly with the aging of the population and changes in lifestyle. The treatment consists of either antiarrhythmic drug therapy or catheter ablation or both. While it is believed that the benefits of radiofrequency ablation (RFA) generally outweigh the risks in properly selected patients; however, RFA is associated with a risk of complications leading to an increase in morbidity and mortality, increased in-hospital length of stay, and a substantial increase in healthcare costs. The risk of RFA postoperative complications continues to be a cause of concern and accurate forecasts of postoperative complications could be useful to both physicians and patients. In patients undergoing AF ablation, a preoperative assessment of the procedural risks and outcomes should be undertaken and this was recommended by the recent AF guidelines [3]^.^

Although preoperative assessment of the risks of the RFA procedure had been widely studied, there were still some limitations to clinical application. First, some studies have revealed risk factors for complications, but their results were inconsistent [4–10]. Thus, there remains a need to further investigate the risk factors. Second, in one study [11] of the limited predictive model, the researchers built a model with limited variables, but were lack of many important factors: including the type of AF, peri-procedural medicine, echocardiography, and laboratory test results. Due to the lack of clinical factors, its reported AUC of any complications after RFA was 0.65 (95% CI = 0.63-0.67) for the derivation cohort and 0.64 (95% CI = 0.62-0.66) for the validation cohort, which was far from satisfactory.

To overcome the above-mentioned challenges, this study first collected a wide range of clinical factors, then define in-hospital complications as clinical outcomes. Finally, as a real-world observational study, this study may provide new evidence to relieve the inconsistency of reported risk factors. The objective of this study was to use machine learning techniques to develop an effective risk model for predicting complications after radiofrequency ablation of atrial fibrillation patients and reveal important risk factors based on the developed model.

## Methods

### Study population

This retrospective cohort study took place at a large-scale hospital in East China (Shanghai Chest Hospital, Shanghai, China). Patients who underwent RFA after being diagnosed as AF from April 2018 to October 2021 were eligible for inclusion in this study. Patients who were younger than 18 were excluded. Patients who underwent RFA procedure simultaneous cardiac valve surgery left atrial appendage surgery or pacemaker implantation were excluded. Patients who had more than one hospital visits of RFA procedure during the study period were treated as multiple samples in this cohort, meaning each RFA procedure of one patient was independent from other RFA procedures of the same patients. A total of 3365 procedures were analyzed in the present study. This study was approved by the Ethics Committee of Shanghai Chest Hospital (Shang, China) with approval number KS (P) 22005. Since the data were collected retrospectively, consent was not required.

### Data collection and definitions

Patients’ demographic characteristics, medical history, signs or symptoms at presentation, electrocardiographic features, laboratory values, and in-hospital clinical outcomes were collected from hospital information systems, laboratory information systems, and electronic health records. For variables with multiple measurements such as heart rate, blood pressure, and other baseline variables like white cell count, and creatinine clearance rate, the last measurements before RFA were collected.

The primary outcome of interest was the occurrence of any complication after RFA, including pulmonary vein stenosis, phrenic nerve injury, periesophageal vagus nerve injury, arteriovenous fistulas/pseudoaneurysm, cardiogenic shock/arrest, cardiac effusion/tamponade, thromboembolic events (ischemic stroke, transient ischemic attack (TIA), peripheral embolism, or pulmonary embolism), pneumothorax, hemorrhage events or myocardial infarction. Hemorrhage events included minor hemorrhages like access site hemorrhage and major hemorrhage. Major hemorrhage events were defined as any bleeding events requiring blood transfusion. Besides, the occurrence of the two most common types of complications, including cardiac effusion/tamponade and hemorrhage, are defined as secondary outcomes.

### Data pre-processing

The data format was unified, duplicates or unmatched items were dropped and outliers were replaced with null values. Q-Q plots, histograms, and Shapiro–Wilk tests were used to assess continuous variable distributions. Outlier was defined as values not lying within 1.5 times the interquartile range from the median. Variables with more than 30% missing values were removed from the analysis. Other variables with equal or less than 30% missing values were imputed by the multivariate imputation by chained equation (MICE) method [12]. The binary variables like gender, drugs use, and medical history were encoded as 0 and 1 (0 = female/no, 1 = male/yes). The model output corresponds to postoperative complication and was represented as a binary class (0 = without complication, 1 = with complication).

### Model construction

We evaluated the prediction performance of the Logistic regression model as well as 4 different machine learning models including decision tree (DT), random forest (RF), gradient boosting machine (GBM), and extreme gradient boosting (XGBoost) that have been demonstrated to apply to medical field and big data sets previously. A total of 59 different features (Table 1) were used as inputs into the prediction models. Multivariable logistic models were fitted using backward stepwise regression. For the stepwise method, Akaike Information Criterion (AIC) was used as the selection criteria to choose the predictors. Moreover, known and potential risk factors such as age or gender were considered in the logistic model. For machine learning models, we applied the grid search method with five-fold cross-validation to identify the optimal hyperparameters, which yield the highest value of AUC.

**Table 1:** Baseline characteristics of patients with or without complications

	**Variables**	**Total (*N* = 3365)**	**Without complications (*N* = 3303)**	**With complications (*N* = 62)**	***P*-value**
Demographic	Gender				0.626
	Male	2108 (62.6)	2071 (62.7)	37 (59.7)	
	Female	1257 (37.4)	1232 (37.3)	25 (40.3)	
	Age Median (IQR)	66 (59,72)	66 (59,72)	71 (64.5,77)	< 0.001
	Height, cm Median (IQR)	168 (160,172)	168 (160,172)	165 (158,170)	0.043
	Weight, kg Median (IQR)	70 (60,76)	70 (60,76)	65.6 (58.5,72.8)	0.02
	BMI, kg/m^2^ Median (IQR)	24.7 (22.8,26.8)	24.7 (22.8,26.8)	24 (22.4,25.9)	0.076
Signs and symptoms at presentation	AF_category n (%)				0.293
	Paroxysmal AF	1512 (48.9)	1492 (49.1)	20 (38.5)	
	Persistent AF	1365 (44.2)	1338 (44)	27 (51.9)	
	Chronic AF	214 (6.9)	209 (6.9)	5 (9.6)	
	HR, bpm Median (IQR)	77 (70,83)	77 (70,83)	74 (68,80)	0.088
	DBP, mmHg Median (IQR)	86 (78,95)	86 (78,95)	82 (73.8,90)	0.01
	SBP, mmHg Median (IQR)	137 (123,150)	137 (123,150)	138 (126.2,147.8)	0.696
	HAS_BLED score Median (IQR)	1 (1,2)	1 (1,2)	2 (1,3)	0.002
	CHA_2DS_2-VACS score Median (IQR)	2 (1,3)	2 (1,3)	3 (2,4)	< 0.001
Echocardiography	LVESD Median (IQR)	29 (27,32)	29 (27,32)	29 (27,30.8)	0.335
	LVEDD Median (IQR)	48 (45,51)	48 (45,51)	47 (44,50)	0.112
	LAD, mm Median (IQR)	42 (37,46)	42 (37,46)	41 (37,46)	0.837
	LVEF Median (IQR)	64 (61,66)	64 (61,66)	63 (60,66)	0.215
Preoperative laboratory values	TSH, mIU/L Median (IQR)	1.8 (1.2,2.7)	1.8 (1.2,2.7)	1.7 (1.1,2.4)	0.597
	FBG, g/L Median (IQR)	2.7 (2.3,3.1)	2.7 (2.3,3.1)	2.9 (2.3,3.1)	0.355
	UA, μmol/L Median (IQR)	358 (299,424)	357 (299,423)	398 (316,462)	0.065
	TT, s Median (IQR)	18.6 (17.6,20)	18.6 (17.6,20)	18.2 (17.2,20)	0.273
	PTINR Median (IQR)	1 (1,1.2)	1 (1,1.2)	1.1 (1,1.2)	0.159
	CREA,μmol/L Median (IQR)	75 (65,87)	75 (64,87)	80 (71,95)	0.021
	Ccr, ml/(min × 1.73m^2^) Median (IQR)	78.6 (63.9,96.6)	78.9 (64.4,96.7)	61.9 (50.2,90.5)	< 0.001
	DD, mg/L Median (IQR)	0.2 (0.2,0.4)	0.2 (0.2,0.4)	0.3 (0.2,0.6)	0.002
	TnI, ng/mL Median (IQR)	0.00 (0.00,0.01)	0.00 (0.00,0.01)	0.01 (0.00,0.02)	< 0.001
	LDH, U/L Median (IQR)	202 (179,232)	202 (179,231)	215 (187,257)	0.036
	AST, U/L Median (IQR)	23 (20,29)	23 (20,29)	25 (20,37)	0.092
	ALB, g/L Median (IQR)	42 (41,44)	42.5 (41,44)	41 (38,44)	0.014
	GLU, mmol/L Median (IQR)	5.9 (5.1,7.3)	5.9 (5.1,7.3)	6.1 (5.2,7.7)	0.257
	CK, U/L Median (IQR)	91 (69,126)	91 (69,126)	88 (69,125)	0.841
	NT-pro-BNP, ng/L Median (IQR)	477 (160,1040)	474.5 (159,1027.5)	749 (282,1735)	0.009
Preoperative drug	Aspirin n (%)	78 (2.3)	76 (2.3)	2 (3.2)	0.632
	Clopidogrel n (%)	83 (2.5)	80 (2.4)	3 (4.8)	0.224
	Other antiplatelet agents n (%)	21 (0.6)	21 (0.6)	0 (0)	0.529
	Antiplatelet agents n (%)	133 (4)	129 (3.9)	4 (6.5)	0.308
	Warfarin n (%)	45 (1.3)	44 (1.3)	1 (1.6)	0.849
	Dabigatran n (%)	194 (5.8)	194 (5.9)	0 (0)	0.049
	Rivaroxaban n (%)	633 (18.8)	616 (18.6)	17 (27.4)	0.08
	Heparin n (%)	2542 (75.5)	2497 (75.6)	45 (72.6)	0.584
	Anticoagulants n (%)	2673 (79.4)	2623 (79.4)	50 (80.6)	0.812
	Statins n (%)	1046 (31.1)	1017 (30.8)	29 (46.8)	0.007
	ACEI/ARB n (%)	578 (17.2)	565 (17.1)	13 (21)	0.424
	β blocker n (%)	786 (23.4)	766 (23.2)	20 (32.3)	0.095
	Diuretics n (%)	1004 (29.8)	981 (29.7)	23 (37.1)	0.207
	CCB n (%)	604 (17.9)	587 (17.8)	17 (27.4)	0.05
	Antihypertensive agents n (%)	1749 (52)	1711 (51.8)	38 (61.3)	0.138
Medical history	Angina n (%)	10 (0.3)	10 (0.3)	0 (0)	0.664
	Heart failure n (%)	17 (0.5)	17 (0.5)	0 (0)	0.571
	Stroke n (%)	419 (12.5)	408 (12.4)	11 (17.7)	0.203
	PAD n (%)	202 (6)	196 (5.9)	6 (9.7)	0.219
	COPD n (%)	75 (2.2)	74 (2.2)	1 (1.6)	0.74
	Hypertension n (%)	1781 (52.9)	1742 (52.7)	39 (62.9)	0.112
	Diabetes n (%)	528 (15.7)	515 (15.6)	13 (21)	0.249
	Hyperlipidemia n (%)	87 (2.6)	85 (2.6)	2 (3.2)	0.748
	MI n (%)	26 (0.8)	26 (0.8)	0 (0)	0.483
	CHD n (%)	410 (12.2)	399 (12.1)	11 (17.7)	0.177
	CKD n (%)	83 (2.5)	80 (2.4)	3 (4.8)	0.224
	Prior RFA n (%)	998 (29.7)	979 (29.6)	19 (30.6)	0.864
	Prior PCI n (%)	160 (4.8)	155 (4.7)	5 (8.1)	0.216
	Prior CABG n (%)	17 (0.5)	17 (0.5)	0 (0)	0.571

*Abbreviations ACEI* Angiotensin-converting enzyme inhibitor, *ALB* Albumin, *ARB* Angiotensin receptor blocker, *AST* Aspartate transaminase, *BMI* Body mass index, *CABG* Coronary artery bypass grafting, *CCB* Calcium channel blocker, *Ccr* Creatinine clearance rate, *CHD* Coronary heart disease, *CK* Creatine kinase, *CKD* Chronic kidney disease, *COPD* Chronic obstructive pulmonary disease, *CREA* Creatinine, *DD* D-dimer, *DBP* Diastolic blood pressure, *FBG* Fibrinogen, *GLU* Glucose, *HR* Heart rate, *LAD* Left atrial diameter, *LDH* Lactate dehydrogenase, *LVEF* Left ventricular ejection fraction, *LVEDD* Left ventricular end diastolic diameter, *LVESD* Left ventricular end systolic diameter, *MI* Myocardial infarction, *NT-pro-BNP* N-terminal pro-B-type natriuretic peptide, *PAD* Peripheral artery disease, *PCI* Percutaneous coronary intervention, *PTINR* International normalized ratio, *RFA* Radiofrequency ablation, *SBP* Systolic blood pressure, *TSH* Thyroid-stimulating hormone, *TnI* Troponin I, *TT* Thrombin time, *UA* Uric acid

### Model evaluation

The performance and estimation of the general error of the models were assessed using 20 times repeated fivefold cross-validation, where the data set is divided into 5 equal parts. In each repetition, one of the 5 parts is used as a test set, while the remaining 4 parts are used as a training set to train the model. The performance of the model is evaluated on the test set, and the process is repeated until each part has been used as the test once. This procedure is repeated for a total of 20 times, with a different random seed used for each repetition to ensure the variability of the results. The final evaluation of the model is based on the average performance across all repetitions. Model discrimination was assessed using the area under the receiver operating characteristic curve (AUC). In addition, we calculated accuracy, sensitivity (recall), specificity, and F score with a cut-off point, which was estimated using the maximized Youden index in the training set. Model calibration was tested by the Brier score. The smaller the Brier score is, the better calibration will be. 95% confidence intervals were calculated by 20 times repeated fivefold cross-validation for each metric. Shapley additive explanations (SHAP) were used to evaluate the importance of variables [13].

### Feature ranking and selection

We used all candidate features to build the initial model. For ease of interpretation and application, machine learning models with top 5, top 10, top 15, and top 20 features were constructed according to the ranked importance of the features. For each machine learning algorithm, the feature subset generating the highest AUC was selected as the optimal feature subset.

### Statistical analysis

Data were presented as the mean ± standard deviation (SD) for normally distributed data, or medians and interquartile range (IQR) for non-normally distributed data. Normally distributed variables were compared using Student’s t-test and non-normally distributed variables were compared using the Mann–Whitney U test. Categorical data were expressed as numbers and percentages (%). Pearson’s χ2 test or Fisher’s exact test were used for categorical data, as appropriate. All *P* values were two-tailed, and a *P*-value of < 0.05 was considered to represent statistical significance. Statistical analysis was performed in R version 4.1.2 and Python 3.9.13. The model development, evaluation, and calibration were performed using the Scikit-learn (1.0.2) and xgboost package (1.7.4) in Python. SHAP values were computed and visualized with the shap package (0.41.0). The imputation was performed in R using package “mice” (3.15.0). The sample data and code are publicly available on the project GitHub website at https://github.com/awei1234/Machine-Learning-Based-Risk-Models-for-Procedural-Complications-of-RFA-for-AF-patients.

## Results

### Study sample and procedural complications

Three thousand sixty five consecutive RFA procedures on 3187 AF patients between April 2018 and October 2021 were collected. Supplementary Figure S1 is a flow chart describing the procedure for subject selection. The variables used for model construction and missing rates were shown in supplementary Table S1. The baseline characteristics of the patients and the comparisons between the two groups with or without complications are shown in Table 1. Patients in the complication group were older than those without complications (71 years, IQR 64.5–77 years vs 66 years, IQR 59–72 years). The proportion of male patients in the complication and non-complication groups was 59.7% and 62.7%, respectively. The baseline characteristics of the patients and the comparisons between the two groups with or without cardiac effusion or hemorrhage are shown in supplementary tables S2 and S3. Table 2 displays the specific procedural complications and total complications. There were a total of 62 procedural complications with a rate of 1.84% in the entire cohort. No procedure-related death was observed. Cardiac effusion/tamponade was the most common and accounted for 0.84% of the entire procedures followed by access site hemorrhage or hematoma (0.62%), hemorrhage requiring blood transfusion (0.27%), thromboembolic events (0.12%), cardiogenic shock/arrest (0.06%), arteriovenous fistulas/pseudoaneurysm (0.06%), pneumothorax (0.03%), pulmonary vein stenosis (0.03%), and phrenic nerve injury (0.03%).

**Table 2:** Complicaitons following radiofrequency ablation in the study population

Complications	Overall n (%)
Cardiac effusion/tamponade	28 (0.83%)
Access site hemorrhage/hematoma	21 (0.62%)
Major hemorrhage (any bleeding events requiring blood transfusion)	9 (0.27%)
Thromboembolic events	4 (0.12%)
Cardiogenic shock/arrest	2 (0.06%)
Arteriovenous fistulas/pseudoaneurysm	2 (0.06%)
Pulmonary vein stenosis	1 (0.03%)
Phrenic nerve injury	1 (0.03%)
Pneumothorax	1 (0.03%)
Any complication	62 (1.84%)

### Feature selection and ranking

**Fig. 1:**
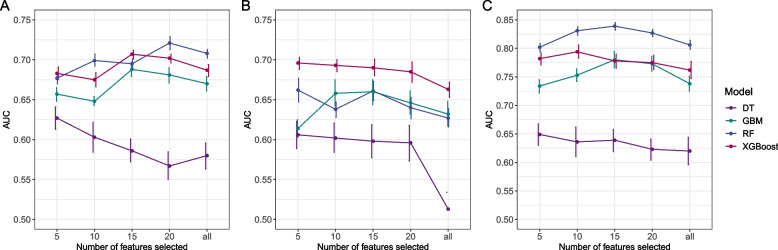
AUC of the model with different numbers of the selected features. **A**: any complication; **B**: cardiac effusion/tamponade; **C**: hemorrhage

When adding features according to their importance, the AUC of DT models consistently decreased (from 0.627 to 0.580 for any complication, from 0.606 to 0.513 for cardiac effusion/tamponade, and from 0.649 to 0.620 for hemorrhage). For postoperative cardiac effusion/tamponade, the GBM model was an exception, showing an increasing trend in AUC with the increase in the number of features. Other machine learning models that used the top 5 ranked features performed better than models with more features. For any complication or hemorrhage, the RF, GBM, and XGBoost models demonstrated good performance, especially when using the top 10, 15 or, 20 features were utilized. The corresponding AUCs were shown in Fig. 1. For any complication, the optimal numbers of features were 5, 20,15, and 15 for DT, RF, GBM, and XGBoost, respectively. For cardiac effusion/tamponade, the optimal numbers of features were 5, 5, 15, and 5. For hemorrhage, the optimal numbers of features were 5, 15,15, and 10. The range of hyper-parameters was shown in supplementary table S4. The evaluation metrics with 95% confidence intervals for each model with different features were shown in supplementary table S5.

### Model performance and comparison

Of the considered machine learning models, the best-performing models were RF for any complication, and cardiac effusion/tamponade, XGBoost for cardiac effusion/tamponade. The AUCs for these models were as follows: 0.721 (95% CI = 0.713–0.729) for any complication, 0.696 (95% CI = 0.688–0.703) for cardiac effusion/tamponade, and 0.839 (95% CI = 0.832–0.845) for hemorrhage.

**Fig. 2:**
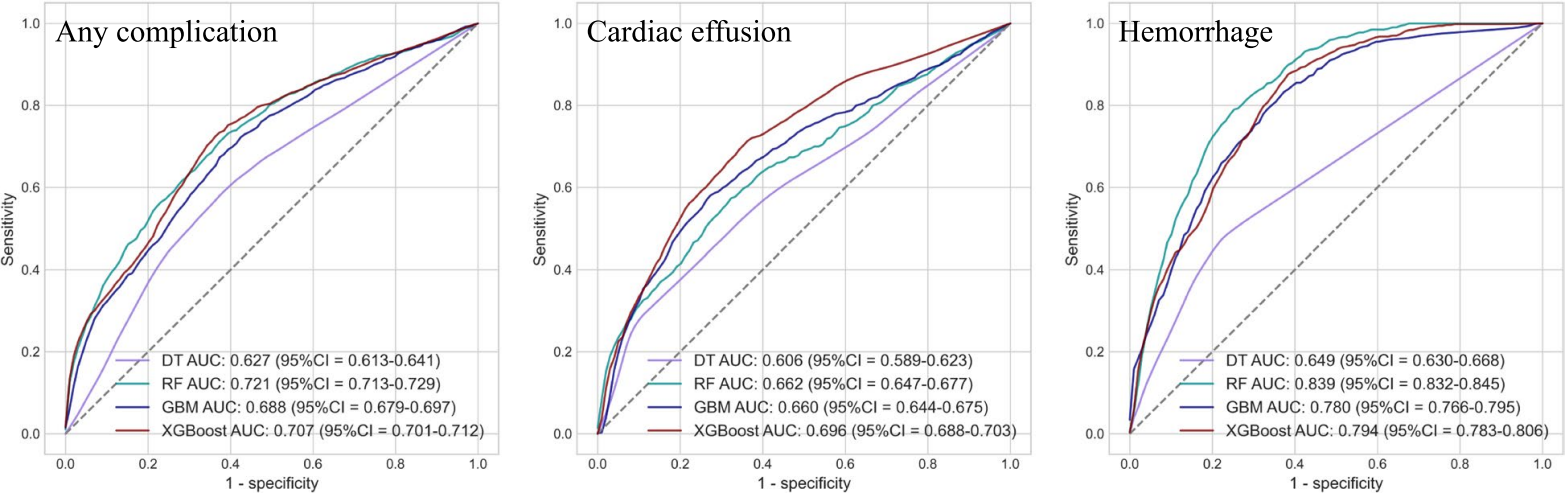
Receiver operating characteristic curves for the DT, RF, GBM, and XGBoost models in predicting any complication, cardiac effusion, and hemorrhage

The receiver operating characteristic (ROC) curves, and performance metrics, including AUC, accuracy, sensitivity, specificity, F score, and Brier score were presented in Fig. 2, and Table 3.

**Table 3:** The evaluation metrics with 95% confidence intervals for each model using 20-round fivefold cross-validation

outcomes	Model	AUC^a^ (95% CI)	Accuracy (95% CI)	Sensitivity (95% CI)	Specificity (95% CI)	F score (95% CI)	Brier score (95% CI)
Any complications	LR^b^	0.650(0.645,0.655)	0.858(0.855,0.860)	0.352(0.339,0.366)	0.867(0.865,0.870)	0.084(0.081,0.087)	0.142(0.140,0.145)
	DT^c^	0.627(0.613,0.641)	0.599(0.584,0.615)	0.615(0.589,0.642)	0.599(0.584,0.615)	0.054(0.051,0.056)	0.401(0.385,0.416)
	RF^d^	0.721(0.713,0.729)	0.834(0.832,0.836)	0.460(0.446,0.475)	0.841(0.838,0.843)	0.092(0.090,0.095)	0.166(0.164,0.168)
	GBM^e^	0.688(0.679,0.697)	0.929(0.927,0.930)	0.239(0.225,0.252)	0.942(0.940,0.943)	0.110(0.103,0.116)	0.071(0.070,0.073)
	XGBoost^f^	0.707(0.701,0.712)	0.899(0.897,0.901)	0.327(0.315,0.340)	0.910(0.908,0.912)	0.107(0.103,0.111)	0.101(0.099,0.103)
Cardiac effusion/tamponade	LR	0.665(0.656,0.674)	0.921(0.918,0.923)	0.259(0.241,0.277)	0.926(0.924,0.929)	0.052(0.048,0.055)	0.079(0.077,0.082)
	DT	0.606(0.589,0.623)	0.429(0.398,0.459)	0.711(0.673,0.749)	0.426(0.396,0.457)	0.020(0.019,0.021)	0.571(0.541,0.602)
	RF	0.662(0.647,0.677)	0.918(0.917,0.919)	0.295(0.281,0.308)	0.923(0.922,0.924)	0.056(0.054,0.059)	0.082(0.081,0.083)
	GBM	0.660(0.644,0.675)	0.945(0.944,0.946)	0.195(0.166,0.223)	0.951(0.950,0.952)	0.055(0.047,0.063)	0.055(0.054,0.056)
	XGBoost	0.696(0.688,0.703)	0.681(0.671,0.692)	0.652(0.632,0.672)	0.682(0.671,0.692)	0.033(0.032,0.034)	0.319(0.308,0.329)
Hemorrhage/hematoma	LR	0.745(0.737,0.752)	0.938(0.936,0.939)	0.207(0.190,0.225)	0.944(0.942,0.945)	0.051(0.046,0.055)	0.062(0.061,0.064)
	DT	0.649(0.630,0.668)	0.807(0.799,0.814)	0.470(0.429,0.512)	0.809(0.801,0.817)	0.037(0.035,0.040)	0.193(0.186,0.201)
	RF	0.839(0.832,0.845)	0.903(0.902,0.904)	0.463(0.440,0.486)	0.906(0.905,0.908)	0.071(0.067,0.075)	0.097(0.096,0.098)
	GBM	0.780(0.766,0.795)	0.985(0.985,0.986)	0.161(0.151,0.171)	0.992(0.991,0.992)	0.148(0.140,0.157)	0.015(0.014,0.015)
	XGBoost	0.794(0.783,0.806)	0.860(0.857,0.862)	0.450(0.428,0.472)	0.863(0.861,0.866)	0.049(0.047,0.051)	0.140(0.138,0.143)

^a^*AUC* Area under the ROC curve

^b^*LR* Logistic regression

^c^*DT* Decision tree

^d^*RF* Random forest

^e^*GBM* Gradient boosting machine

^f^*XGBoost* Extreme gradient boosting

**Fig. 3:**
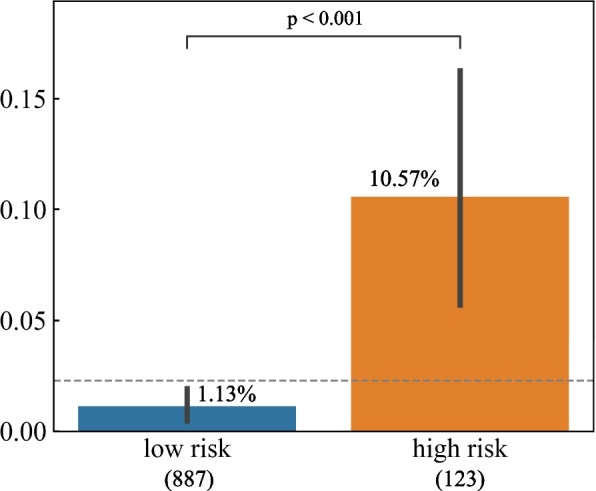
Postoperative complication incidence rate and the number of patients in different risk groups in the test set. Note: The number in brackets, eg. ‘887’ in ‘low risk (887)’ represents the number of patients who are classified into the low-risk group. The grey dashed line represents the actual postoperative complication incidence rate in the test set

To stratify patients into different risk groups, for the RF model the predicted probability of 0–0.029 and > 0.029 were selected to range as low and high risk, respectively. To validate the ability to stratify patients into different risk groups, in Fig. 3, the incidence rate of each risk group and inter-group differences in the test set were compared for the RF model.

### Important features associated with postoperative complications

The result of the logistic regression model postoperative complications is shown in Table 4. Higher values of CREA and AST were associated with increased probabilities of procedural complications. The higher value of AST was associated with increased probabilities of procedural cardiac effusion. Persistent AF, higher values of CREA, DD, and TnI were associated with increased probability of hemorrhage.

**Table 4:** Stepwise multivariable logistic regression model of different outcome

variable	Any complication		Cardiac effusion/tamponade		Hemorrhage	
	**OR (95%CI)**	***P* value**	**OR (95% CI)**	***P* value**	**OR (95% CI)**	***P* value**
Female	0.85 (0.49, 1.44)	0.54	0.91 (0.41, 1.96)	0.82	0.95 (0.41, 2.13)	0.90
Age (< 65 as reference)	1.43 (0.75, 2.87)	0.30	1.27 (0.57, 3.03)	0.56	2.23 (0.85, 6.98)	0.13
BMI (< 25 as reference)	0.76 (0.44, 1.29)	0.32	0.47 (0.19, 1.04)	0.08		
HR (≤ 100 as reference)	0.85 (0.29, 1.98)	0.73				
LAD (normal as reference)	0.70 (0.39, 1.28)	0.24			0.52 (0.21, 1.33)	0.16
CHA2DS2_VACS score (< 2 as reference)	1.73 (0.81, 3.91)	0.17				
CREA (high vs normal or low)	2.44 (1.31, 4.36)	0.00			2.95 (1.24, 6.68)	0.01
TnI (≤ 0.04 ng/mL as reference)	2.23 (0.82, 5.08)	0.08			3.65(1.01, 10.42)	0.03
AST (≤ 40 U/L as reference)	2.29 (1.13, 4.32)	0.01	3.53 (1.40, 8.15)	0.00	2.70 (0.95, 6.60)	0.06
NT proBNP (300–900 as reference)						
< 300 ng/L	0.81 (0.39, 1.70)	0.58	1.00 (0.33, 3.16)	0.99		
> 900 ng/L	1.66 (0.88, 3.21)	0.12	2.17 (0.86, 6.19)	0.12		
ALB (> 35 g/L as reference)			2.95 (0.82, 8.27)	0.06		
AF_category (Paroxysmal AF as reference)						
Persistent AF					2.79 (1.11, 7.47)	0.03
Chronic AF					1.69 (0.24, 7.52)	0.53
DD (≤ 0.55 mg/L as reference)					2.66 (1.15, 5.90)	0.02
Antiplatelet agents					2.58 (0.72, 7.27)	0.10
Hypertension					2.06 (0.83, 5.85)	0.14
Diabetes					1.88 (0.77, 4.32)	0.15

LAD: normal: left artrial diameter < 41 mm in men or < 39 mm in women; enlargement: ≥ 41 mm in men or ≥ 39 mm in women; CREA: low or normal: ≤ 111 μmol/L in men or ≤ 81 μmol/L in women; high: > 111 μmol/L in men or > 81 μmol/L in women

**Fig. 4:**
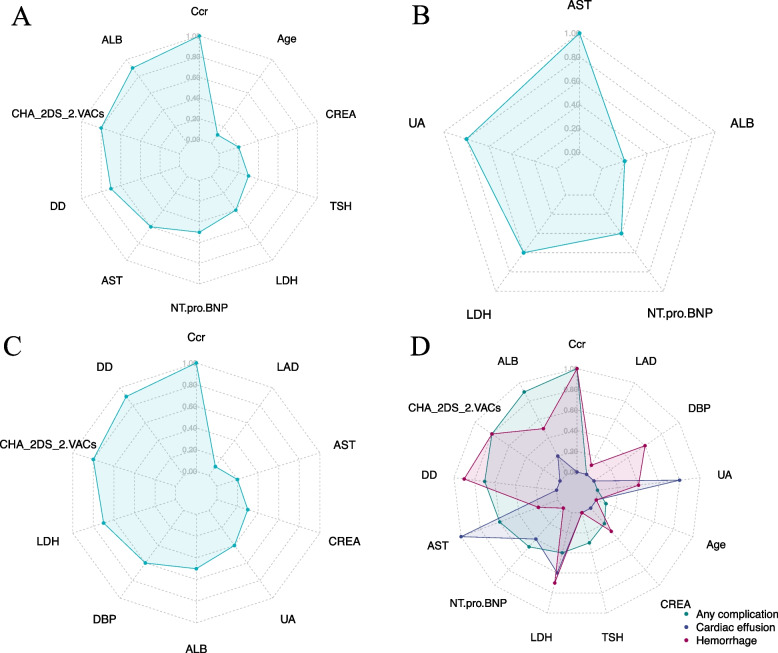
Top-ranked features in predicting different complications. **A**: Top-ranked 10 features derived from the RF model in predicting any complication; **B**: Top-ranked 5 features derived from the XGBoost model in predicting cardiac effusion/tamponade; **C**: Top-ranked 10 features derived from the RF model in predicting hemorrhage; **D**: 13 important features in predicting any complication, cardiac effusion/tamponade or hemorrhage

Based on the RF, GBM, and XGBoost models, the important features among different outcomes have a high degree of coincidence (Fig S2). From the results of the best algorithm models with different outcomes, it is known that the most important risk factors are: Ccr, ALB, CHA_2DS_2-VACs, DD, AST, NT-pro-BNP, LDH, TSH, CREA, age, UA, DBP, and LAD for any complication, cardiac effusion/tamponade or hemorrhage (Fig. 4). In the SHAP summary plots (Fig S3), the distribution of SHAP value contributions is shown for the top-ranked features present in models for predicting different outcomes.

**Fig. 5:**
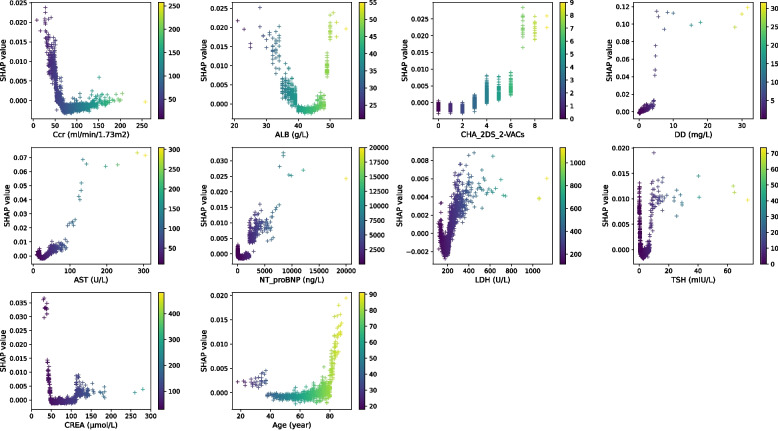
SHAP dependence plot of the RF model in predicting any complication. It shows how a single feature (the top 10 important features) affects the output of the RF model. SHAP values for specific features exceed zero, representing an increasing risk of postoperative complication

Figure 5 shows the SHAP dependence plot of the top 10 most important features for any complication, showing that higher CHA_2DS_2-VACs score, DD, AST, NT-pro-BNP, LDH, age, and lower Ccr, CREA were related to increased risk of any complication. An obvious U-shaped relationship exists between ALB or TSH and the risk of postoperative complication, as both too low and too high levels of ALB or TSH were associated with an increased risk.

From outside to inside, the importance of the feature was successively decreased.

## Discussion

The present study included 3187 patients undergoing RFA (3365 procedures) in a large center that captured real-world clinical information and was used to develop a risk model for complications associated with the procedure.

In this study, the most common complication was cardiac effusion or tamponade (0.83%), similar to the results ranging from 0.5% ~ 1.3% previously reported [8, 14–19]. For vascular complications, previous studies reported incidences from 1.1% to 2.3% [16–18, 20, 21]. In our study, the incidence of access site hemorrhage was 0.62%; hemorrhage requiring transfusion, 0.27%; thromboembolic events, 0.12%; arteriovenous fistulas/pseudoaneurysm, 0.06%; and pulmonary vein stenosis, 0.03%. The overall rate of procedural complications in this study was 1.84%, which is a lower level compared to the complication rates previously reported ranging from 3.3%-6.84% [7, 8, 15–27] to as high as 9.1% [28] in a survey of U.S. medicare patients. Several potential reasons were contributing to the low incidence of postoperative complications in this study. First, we excluded patients undergoing concomitant other surgeries like left atrial appendage closure, leading to a lower incidence of postoperative complications. Second, this study was conducted at a high-volume center, with more than 1000 RFA procedures performed annually. Complication risk was reduced when the surgery occurred in hospitals with high surgery volumes, similar to those reported previously [14, 21, 24]. Finally, the outcome of this study was only based on the in-hospital data.

Using 20 variables identified by machine learning techniques, we developed a predictive model for postoperative complications with good predictive power in AF patients undergoing RFA. According to the definition of the literature [29, 30], the AUC value between 0.7 and 0.8 is acceptable. The model shows better performance (AUC = 0.721) than the model reported previously [11] (AUC = 0.64) and has the potential to be used in clinical practice, particularly for the outcome of hemorrhage, where the AUC reaches 0.839. To evaluate the clinical applicability of the model, patients was stratified into high-risk and low-risk groups according to the probability of the best performed machine learning model. The incidence of postoperative complications difference between two groups was statistically significant.

This study not only developed a more accurate risk model and identified previously unrecognized important risk factors but also made it “explainable”. Our study benefits from the utilization of SHAP values to unveil the “black box” of machine learning models, thus, our model can furnish implications for patient management even when implemented on individual patients. We employed radar plot and as well as SHAP dependence plot for visualized at the feature and the individual level. Among the 10 most important features, most had an obvious cut-point at which the predicted risk abruptly changed. For example, Ccr < 50 ml/(min × 1.73m^2^), ALB > 50 g/L or < 35 g/L, CHA_2DS_2-VACs score ≥ 4, DD > 5 mg/L, AST > 100 U/L, NT-pro-BNP > 2000 ng/L, CREA < 50 μmol/L, or older than 80 resulted in a significant increase in postoperative complication risk.

Ccr is accepted as the best overall measurement for assessing renal function [31], a Ccr < 60 ml/(min × 1.73m^2^) is considered compromised renal function. From the shap dependence plot, reduction of Ccr is shown to increase the risk of postoperative complication, which is consistent with previous research fundings [7, 14]. In our study, ALB is another key predictor for postoperative complication. An obvious U-shaped relationship exists between ALB and the risk of postoperative complication, as both lower than 35 and higher than 50 g/L were associated with an increased risk. Serum ALB is usually used to reflect nutritional status and the ability of the liver to synthesize protein. Decrease in ALB level is indicative liver damage or malnutrition. Meanwhile, several novel findings have been disclosed in our study. Preoperative elevated D-dimer was essential predictors of postoperative complications. Elevated D-dimer indicate a hypercoagulable state and secondary fibrinolysis, which may result in thrombotic disease [32, 33]. Whereas thromboembolic events were infrequent in this study, this could be due to the relatively short length of postoperative hospital stay. Patients with postoperative complications were at a hypercoagulable state at the early stage after ablation procedure but have not yet shown thromboembolic symptoms. Furthermore, preoperative elevated AST, and NT-pro-BNP were essential predictors of postoperative complications in our study. Patients with more comorbidities are more likely to exhibit dysregulated hepatic function, or myocardial function and significantly higher AST, or NT-pro-BNP levels.

The independent factors of procedural complications that have been reported previously were the gender of female [11, 15, 17, 18, 24, 25], older age [11, 16, 20, 24, 25], longer procedural duration [18, 34], the complexity of the procedure [20], CHA_2DS_2-VASc score [8, 9], smaller left atrium dimension [34], and comorbidities like congestive heart failure [11, 16], renal insufficiency [7, 14], coagulopathy [11], peripheral vascular disease [9, 11], chronic obstructive pulmonary disease [11], hypertension [14], mild liver disease [14], diabetes with chronic complications [14], and coronary artery disease [26]. Risk factors like CHA_2DS_2-VACs score, CREA, Ccr, and older age, which are in accordance with previous studies, play an essential role in our model. The inconsistencies between our findings and previous studies are primarily due to the following reasons. Firstly, the differences between studies could result from differences in inclusion criteria or the number of subjects enrolled. Secondly, previous studies mostly included limited variables and included few laboratory indicators. Compared to comorbidities or prior diseases, laboratory indicators for short-term outcome prediction were more objective and sensitive.

To reduce the risk of postoperative complications for AF patients requiring RFA, it is recommended to take the following measures. Firstly, preoperative comprehensive assessment and optimal control of correctable risk factors such as coagulation capability or renal function should be effectively and efficiently implemented in advance to achieve better outcomes. Secondly, the patient’s vital signs and cardiac function throughout the procedure should be closely monitored. Finally, for patients with high risk after RFA, appropriate postoperative care or surveillance is necessary for detecting early complications. Additionally, schedule regular follow-up visits for discharged patients are recommended to assess the patient’s recovery and to provide cardiac rehabilitation and health education.

This study provides additional evidence that can contribute to further research in this field. In this retrospective study, we developed and evaluated different machine learning algorithms using a wide range of features to predict postoperative complications of RFA. Considering the composite outcome of any complication, we conducted sub-models of the most common complication to investigate whether the predictors were different between those two groups. Moreover, for any complication, cardiac effusion, or hemorrhage, over half of the top 10 features were laboratory features. This study demonstrated that the laboratory features, which instantly reflect physical conditions and have been ignored by previous studies, may be more sensitive and more relevant to postoperative complication prediction. One of the advantages of this finding is that it uses variables that are easily accessible within the electronic medical records (EMR). As a result, the model can be integrated into a decision support system under the EMR framework. In practice, this decision support system would access the clinical information of a new patient and calculate the risk of the patient experiencing a postoperative complication.

The present study also has several limitations. Firstly, generalizability is a potential limitation because all patients were included in a single center. Although 3365 procedures were included in this study, with the data collected for patients who presented between 2018 and 2021, the data from a single center, which could not represent the population of Chinese RFA patients, a multi-center study is needed to validate this result. Secondly, this was an in-hospital outcome prediction study based on retrospective use of electronic medical record data, the complications that are known to occur late such as atrio-esophageal fistula might not be captured. The complication rate might be underestimated. However, the majority of the complications occurred in a short period after the RFA procedure, so it is unlikely that a significant number of complications were missed. Finally, although we have included more variables than in previous studies, potential factors such as ablation duration and other intraoperative variables were not available in our database.

## Conclusions

We report an overall complication rate of 1.84% in a large data set of AF radiofrequency ablation. This study indicates that machine learning based on the RF, and XGBoost algorithms showed good performance in predicting different complications after RFA. The model developed in this study may assist clinicians in assessing the risk of complications for patients with AF.

### Supplementary Information

**Additional file 1: Figure S1.** Flow chart of patient selection. **Table S1.** Missing rates of variables. **Table S2.** Baseline characteristics of patients with or without cardiac effusion/tamponade. **Table S3.** Baseline characteristics of patients with or without hemorrhage. **Table S4.** Hyper-parameters of machine learning models. **Table S5.** The evaluation metrics with 95% confidence intervals for each model with different features using 20-round 5-fold cross-validation. **Figure S2.** Top ranked features derived from three machine learning models. **Figure S3.** SHAP summary plot of the machine learning models for different outcomes. A: SHAP summary plot of the top 10 features of the RF model in predicting any complication; B: SHAP summary plot of the top 5 features of the XGBoost model in predicting cardiac effusion/tamponade; C: SHAP summary plot of the top 10 features of the RF model in predicting hemorrhage.

## Data Availability

The data that support the findings of this study are available from Shanghai Chest Hospital but restrictions apply to the availability of these data, which were used under license for the current study, and so are not publicly available. Data are however available from the authors upon reasonable request and with permission of the Ethics Committee of Shanghai Chest Hospital.
