# Supplementary material for: Machine learning-based risk models for procedural complications of radiofrequency ablation for atrial fibrillation
Source: BMC Med Inform Decis Mak. 2023 Nov 10;23:257. doi: 10.1186/s12911-023-02347-5 (PMC10636945; doi:10.1186/s12911-023-02347-5)
Supplement: Supplementary file 1 — Additional file 1: Figure S1. Flow chart of patient selection. Table S1. Missing rates of variables. Table S2. Baseline characteristics of patients with or without cardiac effusion/tamponade. Table S3. Baseline characteristics of patients with or without hemorrhage. Table S4. Hyper-parameters of machine learning models. Table S5. The evaluation metrics with 95% confidence intervals for each model with different features using 20-round 5-fold cross-validation. Figure S2. Top ranked features derived from three machine learning models. Figure S3. SHAP summary plot of the machine learning models for different outcomes. A: SHAP summary plot of the top 10 features of the RF model in predicting any complication; B: SHAP summary plot of the top 5 features of the XGBoost model in predicting cardiac effusion/tamponade; C: SHAP summary plot of the top 10 features of the RF model in predicting hemorrhage. [file 12911_2023_2347_MOESM1_ESM.docx]

**Supplementary Content**

**Figure S1.** Flow chart of patient selection.

**Table S1.** Missing rates of variables.

**Table S2.** Baseline characteristics of patients with or without cardiac effusion/tamponade.

**Table S3.** Baseline characteristics of patients with or without hemorrhage.

**Table S4.** Hyper-parameters of machine learning models.

**Table S5.** The evaluation metrics with 95% confidence intervals for each model with different features using 20-round 5-fold cross-validation.

**Figure S2.** Top ranked features derived from three machine learning models.

**Figure S3.** SHAP summary plot of the machine learning models for different outcomes. A: SHAP summary plot of the top 10 features of the RF model in predicting any complication; B: SHAP summary plot of the top 5 features of the XGBoost model in predicting cardiac effusion/tamponade; C: SHAP summary plot of the top 10 features of the RF model in predicting hemorrhage.

**Figure S1 Flow chart of patient selection**

**Table S1: Missing rates of variables**

|  | **Variables** | **Missing values** | **Missing rate (%)** |
| --- | --- | --- | --- |
| Demographic | Gender | 0 | 0.0 |
|  | Age | 0 | 0.0 |
|  | Height, cm | 344 | 9.4 |
|  | Weight, kg | 342 | 9.3 |
|  | BMI, kg/m^2^ | 346 | 9.4 |
| Signs and symptoms at presentation | AF_category | 442 | 12.1 |
|  | HR, bpm | 3 | 0.0 |
|  | DBP, mmHg | 7 | 0.1 |
|  | SBP, mmHg | 7 | 0.1 |
|  | HAS_BLED score | 654 | 17.8 |
|  | CHA_2DS_2-VACs score | 660 | 18.0 |
| Echocardiography | LVESD | 465 | 12.7 |
|  | LVEDD | 479 | 13.1 |
|  | LAD, mm | 467 | 12.8 |
|  | LVEF | 971 | 26.6 |
| Preoperative laboratory values | TSH, mIU/L | 718 | 19.6 |
|  | FBG, g/L | 56 | 1.5 |
|  | UA, μmol/L | 53 | 1.4 |
|  | TT, s | 54 | 1.4 |
|  | PTINR | 54 | 1.4 |
|  | CREA, μmol/L | 53 | 1.4 |
|  | Ccr, ml/(min×1.73m2) | 392 | 10.7 |
|  | DD, mg/L | 60 | 1.6 |
|  | TnI, ng/mL | 536 | 14.6 |
|  | LDH, U/L | 53 | 1.4 |
|  | AST, U/L | 46 | 1.2 |
|  | ALB, g/L | 56 | 1.5 |
|  | GLU, mmol/L | 258 | 7.0 |
|  | CK, U/L | 61 | 1.6 |
|  | NT-pro-BNP, ng/L | 821 | 22.4 |
| Preoperative drug therapy | Aspirin | 0 | 0.0 |
|  | Clopidogrel | 0 | 0.0 |
|  | Other.antiplatelet agents | 0 | 0.0 |
|  | Antiplatelet agents | 0 | 0.0 |
|  | Warfarin | 0 | 0.0 |
|  | Dabigatran | 0 | 0.0 |
|  | Rivaroxaban | 0 | 0.0 |
|  | Heparin | 0 | 0.0 |
|  | Anticoagulants | 0 | 0.0 |
|  | Statins | 0 | 0.0 |
|  | ACEI/ARB | 0 | 0.0 |
|  | β blocker | 0 | 0.0 |
|  | Diuretics | 0 | 0.0 |
|  | CCB | 0 | 0.0 |
|  | Antihypertensive agents | 0 | 0.0 |
| Medical history | Angina | 0 | 0.0 |
|  | Heart failure | 0 | 0.0 |
|  | Stroke | 0 | 0.0 |
|  | PAD | 0 | 0.0 |
|  | COPD | 0 | 0.0 |
|  | Hypertension | 0 | 0.0 |
|  | Diabetes | 0 | 0.0 |
|  | Hyperlipidemia | 0 | 0.0 |
|  | MI | 0 | 0.0 |
|  | CHD | 0 | 0.0 |
|  | CKD | 0 | 0.0 |
|  | Prior RFA | 0 | 0.0 |
|  | Prior PCI | 0 | 0.0 |
|  | Prior CABG | 0 | 0.0 |

BMI: body mass index; HR: heart rate ; DBP: diastolic blood pressure; SBP: systolic blood pressure; LVESD: Left ventricular end systolic diameter; LVEDD: left ventricular end diastolic diameter; LAD: left atrial diameter; LVEF: left ventricular ejection fraction; PAD: peripheral artery disease; COPD: chronic obstructive pulmonary disease; MI: myocardial infarction; CHD: coronary heart disease; CKD: chronic kidney disease; RFA: radiofrequency ablation; PCI: percutaneous coronary intervention; CABG: coronary artery bypass grafting; ACEI: angiotensin converting enzyme inhibitor; ARB: angiotensin receptor blocker; CCB: calcium channel blocker; TSH: thyroid stimulating hormone; FBG: fibrinogen; UA: uric acid; TT: thrombin time; PTINR: international normalized ratio; CREA: creatinine; Ccr: creatinine clearance rate; DD: D-dimer; LDH: lactate dehydrogenase; ALB: albumin; TnI: Troponin I; AST: aspartate transaminase; GLU: Glucose; CK: creatine kinase; NT-proBNP: N-terminal pro-B-type natriuretic peptide.

**Table S2 Baseline characteristics of patients with or without cardiac effusion/tamponade**

|  | **Variables** | **Without cardiac effusion/tamponade (N=3337)** | **With cardiac effusion/tamponade (N=28)** | **P value** |
| --- | --- | --- | --- | --- |
| Demographic | Gender |  |  | 0.832 |
|  | Male | 2091 (62.7) | 17 (60.7) |  |
|  | Female | 1246 (37.3) | 11 (39.3) |  |
|  | Age | 66 (59,72) | 70.5 (63,76) | 0.014 |
|  | Height, cm | 168 (160,172) | 165.5 (158.5,174) | 0.545 |
|  | Weight, kg | 70 (60,76) | 66.3 (60,72.5) | 0.109 |
|  | BMI, kg/m^2^ | 24.7 (22.8,26.8) | 23.6 (22.5,25) | 0.058 |
| Signs and symptoms at presentation | AF_category |  |  | 0.242 |
|  | Paroxysmal AF | 1504 (49) | 8 (33.3) |  |
|  | Persistent AF | 1352 (44.1) | 13 (54.2) |  |
|  | Chronic AF | 211 (6.9) | 3 (12.5) |  |
|  | HR, bpm | 77 (70,83) | 74 (68,78.2) | 0.147 |
|  | DBP, mmHg | 86 (78,95) | 86 (75.5,97.5) | 0.774 |
|  | SBP, mmHg | 137 (123,150) | 138.5 (129.5,148.5) | 0.75 |
|  | HAS_BLED score | 1 (1,2) | 2 (1,2.5) | 0.143 |
|  | CHA_2DS_2-VACs score | 2 (1,3) | 3 (2,4) | 0.031 |
| Echocardiography | LVESD | 29 (27,32) | 29 (27.2,30.8) | 0.848 |
|  | LVEDD | 48 (45,51) | 48 (46,51) | 0.974 |
|  | LAD, mm | 42 (37,46) | 43 (37.5,47.5) | 0.567 |
|  | LVEF | 64 (61,66) | 63 (60,66) | 0.335 |
| Preoperative laboratory values | TSH, mIU/L | 1.8 (1.2,2.7) | 1.9 (1.2,2.6) | 0.848 |
|  | FBG, g/L | 2.7 (2.3,3.1) | 2.6 (2.2,3.1) | 0.923 |
|  | UA, μmol/L | 358 (300,424) | 323 (265,438.5) | 0.468 |
|  | TT, s | 18.6 (17.6,20) | 18.8 (18,20.9) | 0.539 |
|  | PTINR | 1 (1,1.2) | 1 (1,1.2) | 0.433 |
|  | CREA, μmol/L | 75 (65,87) | 75 (62,88) | 0.826 |
|  | Ccr, ml/(min×1.73m^2^) | 78.7 (64.1,96.6) | 69.3 (58.2,95.5) | 0.301 |
|  | DD, mg/L | 0.2 (0.2,0.4) | 0.3 (0.2,0.5) | 0.068 |
|  | TnI, ng/mL | 0 (0,0) | 0 (0,0) | 0.006 |
|  | LDH, U/L | 202 (179,231) | 207 (194,241) | 0.194 |
|  | AST, U/L | 23 (20,29) | 29 (22.5,44.5) | 0.004 |
|  | ALB, g/L | 42 (41,44) | 41 (38,43) | 0.007 |
|  | GLU, mmol/L | 5.9 (5.1,7.3) | 6 (5,6.9) | 0.979 |
|  | CK, U/L | 91 (69,126) | 83 (69,128.5) | 0.899 |
|  | NT-pro-BNP, ng/L | 476 (160,1030) | 1310 (293.2,2170) | 0.019 |
| Preoperative drug therapy | Aspirin |  |  | 0.413 |
|  | No | 3259 (97.7) | 28 (100) |  |
|  | Yes | 78 (2.3) | 0 (0) |  |
|  | Clopidogrel |  |  | 0.398 |
|  | No | 3254 (97.5) | 28 (100) |  |
|  | Yes | 83 (2.5) | 0 (0) |  |
|  | Other.antiplatelet agents |  |  | 0.674 |
|  | No | 3316 (99.4) | 28 (100) |  |
|  | Yes | 21 (0.6) | 0 (0) |  |
|  | Antiplatelet agents |  |  | 0.281 |
|  | No | 3204 (96) | 28 (100) |  |
|  | Yes | 133 (4) | 0 (0) |  |
|  | Warfarin |  |  | 0.301 |
|  | No | 3293 (98.7) | 27 (96.4) |  |
|  | Yes | 44 (1.3) | 1 (3.6) |  |
|  | Dabigatran |  |  | 0.189 |
|  | No | 3143 (94.2) | 28 (100) |  |
|  | Yes | 194 (5.8) | 0 (0) |  |
|  | Rivaroxaban |  |  | 0.184 |
|  | No | 2712 (81.3) | 20 (71.4) |  |
|  | Yes | 625 (18.7) | 8 (28.6) |  |
|  | Heparin |  |  | 0.947 |
|  | No | 816 (24.5) | 7 (25) |  |
|  | Yes | 2521 (75.5) | 21 (75) |  |
|  | Anticoagulants |  |  | 0.91 |
|  | No | 686 (20.6) | 6 (21.4) |  |
|  | Yes | 2651 (79.4) | 22 (78.6) |  |
|  | Statins |  |  | 0.078 |
|  | No | 2304 (69) | 15 (53.6) |  |
|  | Yes | 1033 (31) | 13 (46.4) |  |
|  | ACEI/ARB |  |  | 0.549 |
|  | No | 2765 (82.9) | 22 (78.6) |  |
|  | Yes | 572 (17.1) | 6 (21.4) |  |
|  | β blocker |  |  | 0.27 |
|  | No | 2560 (76.7) | 19 (67.9) |  |
|  | Yes | 777 (23.3) | 9 (32.1) |  |
|  | Diuretics |  |  | 0.13 |
|  | No | 2345 (70.3) | 16 (57.1) |  |
|  | Yes | 992 (29.7) | 12 (42.9) |  |
|  | CCB |  |  | 0.63 |
|  | No | 2739 (82.1) | 22 (78.6) |  |
|  | Yes | 598 (17.9) | 6 (21.4) |  |
|  | Antihypertensive agents |  |  | 0.091 |
|  | No | 1607 (48.2) | 9 (32.1) |  |
|  | Yes | 1730 (51.8) | 19 (67.9) |  |
| Medical history | Angina |  |  | 0.772 |
|  | No | 3327 (99.7) | 28 (100) |  |
|  | Yes | 10 (0.3) | 0 (0) |  |
|  | Heart failure |  |  | 0.705 |
|  | No | 3320 (99.5) | 28 (100) |  |
|  | Yes | 17 (0.5) | 0 (0) |  |
|  | Stroke |  |  | 0.78 |
|  | No | 2921 (87.5) | 25 (89.3) |  |
|  | Yes | 416 (12.5) | 3 (10.7) |  |
|  | PAD |  |  | 0.799 |
|  | No | 3137 (94) | 26 (92.9) |  |
|  | Yes | 200 (6) | 2 (7.1) |  |
|  | COPD |  |  | 0.422 |
|  | No | 3262 (97.8) | 28 (100) |  |
|  | Yes | 75 (2.2) | 0 (0) |  |
|  | Hypertension |  |  | 0.227 |
|  | No | 1574 (47.2) | 10 (35.7) |  |
|  | Yes | 1763 (52.8) | 18 (64.3) |  |
|  | Diabetes |  |  | 0.467 |
|  | No | 2812 (84.3) | 25 (89.3) |  |
|  | Yes | 525 (15.7) | 3 (10.7) |  |
|  | Hyperlipidemia |  |  | 0.127 |
|  | No | 3252 (97.5) | 26 (92.9) |  |
|  | Yes | 85 (2.5) | 2 (7.1) |  |
|  | MI |  |  | 0.639 |
|  | No | 3311 (99.2) | 28 (100) |  |
|  | Yes | 26 (0.8) | 0 (0) |  |
|  | CHD |  |  | 0.733 |
|  | No | 2931 (87.8) | 24 (85.7) |  |
|  | Yes | 406 (12.2) | 4 (14.3) |  |
|  | CKD |  |  | 0.398 |
|  | No | 3254 (97.5) | 28 (100) |  |
|  | Yes | 83 (2.5) | 0 (0) |  |
|  | Prior RFA |  |  | 0.899 |
|  | No | 2347 (70.3) | 20 (71.4) |  |
|  | Yes | 990 (29.7) | 8 (28.6) |  |
|  | Prior PCI |  |  | 0.551 |
|  | No | 3179 (95.3) | 26 (92.9) |  |
|  | Yes | 158 (4.7) | 2 (7.1) |  |
|  | Prior CABG |  |  | 0.705 |
|  | No | 3320 (99.5) | 28 (100) |  |
|  | Yes | 17 (0.5) | 0 (0) |  |

Abbreviations: ACEI: angiotensin converting enzyme inhibitor; ALB: albumin; ARB: angiotensin receptor blocker; AST: aspartate transaminase; BMI: body mass index; CABG: coronary artery bypass grafting; CCB: calcium channel blocker; CREA: creatinine; Ccr: creatinine clearance rate; CHD: coronary heart disease; CK: creatine kinase; CKD: chronic kidney disease; COPD: chronic obstructive pulmonary disease; CREA: creatinine; DD: D-dimer; DBP: diastolic blood pressure; FBG: fibrinogen; GLU: Glucose; HR: heart rate ; LAD: left atrial diameter; LDH: lactate dehydrogenase; LVEF: left ventricular ejection fraction; LVEDD: left ventricular end diastolic diameter; LVESD: Left ventricular end systolic diameter; MI: myocardial infarction; NT-proBNP: N-terminal pro-B-type natriuretic peptide; PAD: peripheral artery disease; PCI: percutaneous coronary intervention; PTINR: international normalized ratio; RFA: radiofrequency ablation; SBP: systolic blood pressure; TSH: thyroid stimulating hormone; TnI: Troponin I; TT: thrombin time; UA: uric acid.

**Table S3 Baseline characteristics of patients with or without hemorrhage**

|  | **Variables** | **Without hemorrhage (N=3338)** | **With hemorrhage (N=27)** | **P value** |
| --- | --- | --- | --- | --- |
| Demographic | Gender |  |  | 0.715 |
|  | Male | 2092 (62.7) | 16 (59.3) |  |
|  | Female | 1246 (37.3) | 11 (40.7) |  |
|  | Age | 66 (59,72) | 72 (68.5,80) | < 0.001 |
|  | Height, cm | 168 (160,172) | 162 (158,168) | 0.028 |
|  | Weight, kg | 70 (60,76) | 67.7 (60,74) | 0.46 |
|  | BMI, kg/m^2^ | 24.7 (22.8,26.8) | 25.4 (24,26.9) | 0.445 |
| Signs and symptoms at presentation | AF_category |  |  | 0.359 |
|  | Paroxysmal AF | 1505 (49) | 7 (35) |  |
|  | Persistent AF | 1353 (44.1) | 12 (60) |  |
|  | Chronic AF | 213 (6.9) | 1 (5) |  |
|  | HR, bpm | 77 (70,83) | 74 (68,89.5) | 0.823 |
|  | DBP, mmHg | 86 (78,95) | 81 (73.5,86) | 0.001 |
|  | SBP, mmHg | 137 (123,150) | 133 (119.5,148.5) | 0.584 |
|  | HAS_BLED | 1 (1,2) | 2 (2,4) | < 0.001 |
|  | CHA_2DS_2-VACs | 2 (1,3) | 4 (3,5) | < 0.001 |
| Echocardiography | LVESD | 29 (27,32) | 29 (27,29) | 0.077 |
|  | LVEDD | 48 (45,51) | 45.5 (44,48) | 0.027 |
|  | LAD,mm | 42 (37,46) | 41 (37,42) | 0.227 |
|  | LVEF | 64 (61,66) | 65 (63,66) | 0.526 |
| Preoperative laboratory values | TSH, mIU/L | 1.8 (1.2,2.7) | 1.8 (1.2,2.3) | 0.671 |
|  | FBG, g/L | 2.7 (2.3,3.1) | 2.8 (2.1,3.1) | 0.956 |
|  | UA, μmol/L | 357 (299,423) | 416 (358.5,459) | 0.003 |
|  | TT, s | 18.6 (17.6,20) | 18.2 (17.2,20.2) | 0.334 |
|  | PTINR | 1 (1,1.2) | 1.1 (1,1.3) | 0.142 |
|  | CREA, μmol/L | 75 (64,87) | 90 (74.5,109.5) | 0.002 |
|  | Ccr, ml/(min×1.73m^2^) | 78.8 (64.3,96.6) | 56.6 (48.6,65.1) | < 0.001 |
|  | DD, mg/L | 0.2 (0.2,0.4) | 0.4 (0.2,1.2) | 0.003 |
|  | TnI, ng/mL | 0 (0,0) | 0 (0,0) | < 0.001 |
|  | LDH, U/L | 202 (179,231) | 217 (188.5,261.5) | 0.066 |
|  | AST, U/L | 23 (20,29) | 25 (21,38.5) | 0.128 |
|  | ALB, g/L | 42 (41,44) | 41 (38.5,45) | 0.187 |
|  | GLU, mmol/L | 5.9 (5.1,7.3) | 6.2 (5.4,8.1) | 0.248 |
|  | CK, U/L | 91 (69,126) | 93 (68.5,121) | 0.882 |
|  | NT-pro-BNP, ng/L | 475.5 (160,1030) | 737 (313,1350) | 0.133 |
| Preoperative drug therapy | Aspirin |  |  | 0.078 |
|  | No | 3262 (97.7) | 25 (92.6) |  |
|  | Yes | 76 (2.3) | 2 (7.4) |  |
|  | Clopidogrel |  |  | 0.004 |
|  | No | 3258 (97.6) | 24 (88.9) |  |
|  | Yes | 80 (2.4) | 3 (11.1) |  |
|  | Other antiplatelet agents |  |  | 0.679 |
|  | No | 3317 (99.4) | 27 (100) |  |
|  | Yes | 21 (0.6) | 0 (0) |  |
|  | Antiplatelet agents |  |  | 0.004 |
|  | No | 3209 (96.1) | 23 (85.2) |  |
|  | Yes | 129 (3.9) | 4 (14.8) |  |
|  | Warfarin |  |  | 0.544 |
|  | No | 3293 (98.7) | 27 (100) |  |
|  | Yes | 45 (1.3) | 0 (0) |  |
|  | Dabigatran |  |  | 0.197 |
|  | No | 3144 (94.2) | 27 (100) |  |
|  | Yes | 194 (5.8) | 0 (0) |  |
|  | Rivaroxaban |  |  | 0.342 |
|  | No | 2712 (81.2) | 20 (74.1) |  |
|  | Yes | 626 (18.8) | 7 (25.9) |  |
|  | Heparin |  |  | 0.53 |
|  | No | 815 (24.4) | 8 (29.6) |  |
|  | Yes | 2523 (75.6) | 19 (70.4) |  |
|  | Anticoagulants |  |  | 0.458 |
|  | No | 688 (20.6) | 4 (14.8) |  |
|  | Yes | 2650 (79.4) | 23 (85.2) |  |
|  | Statins |  |  | 0.019 |
|  | No | 2306 (69.1) | 13 (48.1) |  |
|  | Yes | 1032 (30.9) | 14 (51.9) |  |
|  | ACEI/ARB |  |  | 0.744 |
|  | No | 2764 (82.8) | 23 (85.2) |  |
|  | Yes | 574 (17.2) | 4 (14.8) |  |
|  | β blocker |  |  | 0.092 |
|  | No | 2562 (76.8) | 17 (63) |  |
|  | Yes | 776 (23.2) | 10 (37) |  |
|  | Diuretics |  |  | 0.214 |
|  | No | 2345 (70.3) | 16 (59.3) |  |
|  | Yes | 993 (29.7) | 11 (40.7) |  |
|  | CCB |  |  | 0.009 |
|  | No | 2744 (82.2) | 17 (63) |  |
|  | Yes | 594 (17.8) | 10 (37) |  |
|  | Antihypertensive agents |  |  | 0.251 |
|  | No | 1606 (48.1) | 10 (37) |  |
|  | Yes | 1732 (51.9) | 17 (63) |  |
| Medical history | Angina |  |  | 0.776 |
|  | No | 3328 (99.7) | 27 (100) |  |
|  | Yes | 10 (0.3) | 0 (0) |  |
|  | Heart failure |  |  | 0.71 |
|  | No | 3321 (99.5) | 27 (100) |  |
|  | Yes | 17 (0.5) | 0 (0) |  |
|  | Stroke |  |  | 0.033 |
|  | No | 2926 (87.7) | 20 (74.1) |  |
|  | Yes | 412 (12.3) | 7 (25.9) |  |
|  | PAD |  |  | 0.614 |
|  | No | 3137 (94) | 26 (96.3) |  |
|  | Yes | 201 (6) | 1 (3.7) |  |
|  | COPD |  |  | 0.602 |
|  | No | 3264 (97.8) | 26 (96.3) |  |
|  | Yes | 74 (2.2) | 1 (3.7) |  |
|  | Hypertension |  |  | 0.009 |
|  | No | 1578 (47.3) | 6 (22.2) |  |
|  | Yes | 1760 (52.7) | 21 (77.8) |  |
|  | Diabetes |  |  | 0.011 |
|  | No | 2819 (84.5) | 18 (66.7) |  |
|  | Yes | 519 (15.5) | 9 (33.3) |  |
|  | Hyperlipidemia |  |  | 0.395 |
|  | No | 3251 (97.4) | 27 (100) |  |
|  | Yes | 87 (2.6) | 0 (0) |  |
|  | MI |  |  | 0.645 |
|  | No | 3312 (99.2) | 27 (100) |  |
|  | Yes | 26 (0.8) | 0 (0) |  |
|  | CHD |  |  | 0.109 |
|  | No | 2934 (87.9) | 21 (77.8) |  |
|  | Yes | 404 (12.1) | 6 (22.2) |  |
|  | CKD |  |  | 0.004 |
|  | No | 3258 (97.6) | 24 (88.9) |  |
|  | Yes | 80 (2.4) | 3 (11.1) |  |
|  | Prior RFA |  |  | 0.396 |
|  | No | 2346 (70.3) | 21 (77.8) |  |
|  | Yes | 992 (29.7) | 6 (22.2) |  |
|  | Prior PCI |  |  | 0.516 |
|  | No | 3180 (95.3) | 25 (92.6) |  |
|  | Yes | 158 (4.7) | 2 (7.4) |  |
|  | Prior CABG |  |  | 0.71 |
|  | No | 3321 (99.5) | 27 (100) |  |
|  | Yes | 17 (0.5) | 0 (0) |  |

Abbreviations: ACEI: angiotensin converting enzyme inhibitor; ALB: albumin; ARB: angiotensin receptor blocker; AST: aspartate transaminase; BMI: body mass index; CABG: coronary artery bypass grafting; CCB: calcium channel blocker; Ccr: creatinine clearance rate; CHD: coronary heart disease; CK: creatine kinase; CKD: chronic kidney disease; COPD: chronic obstructive pulmonary disease; CREA: creatinine; DD: D-dimer; DBP: diastolic blood pressure; FBG: fibrinogen; GLU: Glucose; HR: heart rate; LAD: left atrial diameter; LDH: lactate dehydrogenase; LVEF: left ventricular ejection fraction; LVEDD: left ventricular end diastolic diameter; LVESD: Left ventricular end systolic diameter; MI: myocardial infarction; NT-proBNP: N-terminal pro-B-type natriuretic peptide; PAD: peripheral artery disease; PCI: percutaneous coronary intervention; PTINR: international normalized ratio; RFA: radiofrequency ablation; SBP: systolic blood pressure; TSH: thyroid stimulating hormone; TnI: Troponin I; TT: thrombin time; UA: uric acid.

**Table S4: Hyper-parameters of machine learning models**

| **Model** | **Hyper-parameters** | **value range** | **used for any complication** | **used for cardiac effusion** | **used for hemorrhage** |
| --- | --- | --- | --- | --- | --- |
| DT | criterion | "gini", "entropy", "log_loss" | "entropy" | "entropy" | "gini" |
|  | max_depth | 1, 3, 5, 7, 10, 15 | 5 | 3 | 5 |
|  | min_samples_split | 2, 5, 10, 15, 20, 30, 50 | 2 | 2 | 30 |
|  | min_samples_leaf | 2, 5, 10, 15, 20, 30, 50 | 50 | 50 | 10 |
| RF | n_estimators | 50, 100, 200, 300 | 100 | 100 | 200 |
|  | criterion | "gini", "entropy", "log_loss" | "gini" | "gini" | "entropy" |
|  | max_depth | 3, 5, 7, 10 | 7 | 10 | 7 |
|  | min_samples_leaf | 2, 5, 10, 20, 50 | 5 | 10 | 20 |
|  | min_samples_split | 2, 5, 10, 20, 50 | 50 | 2 | 50 |
| GBM | learning rate | 0.001, 0.005, 0.01, 0.05, 0.1 | 0.01 | 0.01 | 0.01 |
|  | n_estimators | 50, 100, 200, 300 | 100 | 100 | 100 |
|  | max_depth | 3, 5, 7, 10 | 10 | 7 | 7 |
|  | min_samples_split | 2, 5, 10, 20, 50 | 2 | 20 | 20 |
|  | min_samples_leaf | 2, 5, 10, 20, 50 | 2 | 5 | 5 |
|  | subsample | 0.5, 0.7, 0.9 | 0.5 | 0.7 | 0.7 |
| XGBoost | eta | 0.001, 0.005, 0.01, 0.05, 0.1 | 0.005 | 0.05 | 0.05 |
|  | n_estimators | 50, 100, 200, 300 | 300 | 50 | 100 |
|  | max_depth | 3, 5, 7, 10 | 10 | 3 | 3 |
|  | min_child_weight | 3, 5, 10, 15, 20 | 3 | 15 | 10 |
|  | colsample_bytree | 0.5, 0.7, 0.9 | 0.5 | 0.5 | 0.9 |
|  | subsample | 0.5, 0.7, 0.9 | 0.9 | 0.5 | 0.9 |

**Table S5 The evaluation metrics with 95% confidence intervals for each model with different features using 10-round 5-fold cross-validation**

| outcomes | Model | features | AUC (95%CI) | Accuracy (95%CI) | Sensitivity (95%CI) | Specificity (95%CI) |
| --- | --- | --- | --- | --- | --- | --- |
| Any complications | DT | top5 | 0.627(0.613,0.641) | 0.599(0.584,0.615) | 0.615(0.589,0.642) | 0.599(0.584,0.615) |
|  |  | top10 | 0.603(0.584,0.622) | 0.756(0.748,0.764) | 0.465(0.43,0.499) | 0.761(0.753,0.77) |
|  |  | top15 | 0.586(0.572,0.601) | 0.739(0.716,0.762) | 0.41(0.376,0.444) | 0.745(0.721,0.769) |
|  |  | top20 | 0.567(0.55,0.585) | 0.429(0.394,0.465) | 0.663(0.614,0.712) | 0.425(0.388,0.462) |
|  |  | all | 0.58(0.563,0.596) | 0.621(0.586,0.655) | 0.555(0.512,0.597) | 0.622(0.586,0.658) |
|  | RF | top5 | 0.677(0.67,0.683) | 0.805(0.803,0.807) | 0.365(0.352,0.378) | 0.814(0.812,0.816) |
|  |  | top10 | 0.699(0.692,0.707) | 0.865(0.863,0.868) | 0.298(0.287,0.308) | 0.876(0.874,0.878) |
|  |  | top15 | 0.695(0.689,0.701) | 0.835(0.833,0.838) | 0.387(0.375,0.4) | 0.844(0.842,0.846) |
|  |  | top20 | 0.721(0.713,0.729) | 0.834(0.832,0.836) | 0.46(0.446,0.475) | 0.841(0.838,0.843) |
|  |  | all | 0.708(0.702,0.713) | 0.805(0.803,0.807) | 0.514(0.5,0.528) | 0.81(0.808,0.812) |
|  | GBM | top5 | 0.657(0.648,0.665) | 0.918(0.916,0.92) | 0.185(0.17,0.201) | 0.932(0.93,0.934) |
|  |  | top10 | 0.648(0.643,0.654) | 0.909(0.908,0.911) | 0.203(0.19,0.216) | 0.923(0.921,0.925) |
|  |  | top15 | 0.688(0.679,0.697) | 0.929(0.927,0.93) | 0.239(0.225,0.252) | 0.942(0.94,0.943) |
|  |  | top20 | 0.681(0.671,0.69) | 0.936(0.935,0.937) | 0.191(0.172,0.21) | 0.95(0.949,0.951) |
|  |  | all | 0.67(0.661,0.679) | 0.931(0.93,0.932) | 0.207(0.192,0.222) | 0.944(0.943,0.945) |
|  | XGBoost | top5 | 0.683(0.675,0.691) | 0.939(0.938,0.941) | 0.141(0.128,0.154) | 0.954(0.953,0.956) |
|  |  | top10 | 0.675(0.666,0.684) | 0.878(0.876,0.88) | 0.272(0.255,0.288) | 0.889(0.887,0.891) |
|  |  | top15 | 0.707(0.701,0.712) | 0.899(0.897,0.901) | 0.327(0.315,0.34) | 0.91(0.908,0.912) |
|  |  | top20 | 0.702(0.696,0.707) | 0.916(0.914,0.917) | 0.298(0.282,0.313) | 0.927(0.926,0.929) |
|  |  | all | 0.687(0.679,0.694) | 0.921(0.919,0.922) | 0.271(0.26,0.282) | 0.933(0.931,0.934) |
| Cardiac effusion/tamponade | DT | top5 | 0.606(0.589,0.623) | 0.429(0.398,0.459) | 0.711(0.673,0.749) | 0.426(0.396,0.457) |
|  |  | top10 | 0.602(0.584,0.621) | 0.738(0.705,0.771) | 0.423(0.391,0.455) | 0.74(0.707,0.774) |
|  |  | top15 | 0.598(0.577,0.619) | 0.789(0.768,0.811) | 0.379(0.345,0.412) | 0.793(0.771,0.815) |
|  |  | top20 | 0.596(0.573,0.618) | 0.789(0.768,0.811) | 0.371(0.335,0.408) | 0.793(0.771,0.814) |
|  |  | all | 0.513(0.493,0.534) | 0.591(0.561,0.621) | 0.405(0.358,0.453) | 0.593(0.562,0.623) |
|  | RF | top5 | 0.662(0.647,0.677) | 0.918(0.917,0.919) | 0.295(0.281,0.308) | 0.923(0.922,0.924) |
|  |  | top10 | 0.638(0.628,0.647) | 0.93(0.929,0.932) | 0.25(0.235,0.265) | 0.936(0.935,0.938) |
|  |  | top15 | 0.661(0.649,0.673) | 0.909(0.908,0.911) | 0.291(0.268,0.314) | 0.915(0.913,0.916) |
|  |  | top20 | 0.64(0.626,0.653) | 0.902(0.9,0.904) | 0.305(0.277,0.333) | 0.907(0.905,0.909) |
|  |  | all | 0.627(0.616,0.639) | 0.922(0.92,0.924) | 0.229(0.207,0.251) | 0.928(0.926,0.93) |
|  | GBM | top5 | 0.614(0.603,0.625) | 0.971(0.97,0.972) | 0.136(0.118,0.153) | 0.978(0.977,0.979) |
|  |  | top10 | 0.658(0.642,0.675) | 0.969(0.969,0.97) | 0.125(0.109,0.141) | 0.976(0.976,0.977) |
|  |  | top15 | 0.66(0.644,0.675) | 0.945(0.944,0.946) | 0.195(0.166,0.223) | 0.951(0.95,0.952) |
|  |  | top20 | 0.646(0.632,0.661) | 0.945(0.944,0.946) | 0.189(0.163,0.216) | 0.952(0.951,0.953) |
|  |  | all | 0.632(0.617,0.648) | 0.938(0.937,0.939) | 0.184(0.161,0.206) | 0.944(0.943,0.945) |
|  | XGBoost | top5 | 0.696(0.688,0.703) | 0.681(0.671,0.692) | 0.652(0.632,0.672) | 0.682(0.671,0.692) |
|  |  | top10 | 0.693(0.685,0.7) | 0.709(0.701,0.717) | 0.636(0.616,0.656) | 0.71(0.701,0.718) |
|  |  | top15 | 0.69(0.68,0.701) | 0.699(0.693,0.706) | 0.63(0.612,0.649) | 0.7(0.693,0.706) |
|  |  | top20 | 0.685(0.672,0.697) | 0.707(0.703,0.711) | 0.627(0.604,0.649) | 0.708(0.703,0.712) |
|  |  | all | 0.663(0.653,0.672) | 0.661(0.653,0.669) | 0.607(0.585,0.63) | 0.661(0.653,0.67) |
| Hemorrhage/hematoma | DT | top5 | 0.649(0.63,0.668) | 0.807(0.799,0.814) | 0.47(0.429,0.512) | 0.809(0.801,0.817) |
|  |  | top10 | 0.636(0.61,0.662) | 0.833(0.823,0.843) | 0.439(0.39,0.488) | 0.836(0.825,0.846) |
|  |  | top15 | 0.639(0.619,0.658) | 0.77(0.756,0.784) | 0.483(0.439,0.527) | 0.772(0.758,0.787) |
|  |  | top20 | 0.623(0.604,0.641) | 0.837(0.827,0.848) | 0.38(0.334,0.425) | 0.841(0.83,0.852) |
|  |  | all | 0.62(0.596,0.644) | 0.758(0.744,0.772) | 0.469(0.426,0.511) | 0.76(0.747,0.774) |
|  | RF | top5 | 0.802(0.794,0.809) | 0.891(0.89,0.892) | 0.493(0.463,0.522) | 0.894(0.893,0.896) |
|  |  | top10 | 0.831(0.823,0.838) | 0.889(0.887,0.891) | 0.411(0.389,0.434) | 0.893(0.891,0.894) |
|  |  | top15 | 0.839(0.832,0.845) | 0.903(0.902,0.904) | 0.463(0.44,0.486) | 0.906(0.905,0.908) |
|  |  | top20 | 0.827(0.821,0.833) | 0.907(0.906,0.909) | 0.437(0.413,0.461) | 0.911(0.91,0.913) |
|  |  | all | 0.806(0.798,0.814) | 0.914(0.912,0.915) | 0.372(0.353,0.391) | 0.918(0.917,0.919) |
|  | GBM | top5 | 0.734(0.722,0.745) | 0.977(0.976,0.978) | 0.156(0.141,0.17) | 0.983(0.982,0.984) |
|  |  | top10 | 0.753(0.742,0.764) | 0.975(0.974,0.976) | 0.169(0.152,0.185) | 0.982(0.981,0.982) |
|  |  | top15 | 0.78(0.766,0.795) | 0.985(0.985,0.986) | 0.161(0.151,0.171) | 0.992(0.991,0.992) |
|  |  | top20 | 0.773(0.76,0.786) | 0.985(0.985,0.986) | 0.148(0.14,0.156) | 0.992(0.991,0.992) |
|  |  | all | 0.738(0.725,0.751) | 0.985(0.984,0.985) | 0.141(0.13,0.151) | 0.992(0.991,0.992) |
|  | XGBoost | top5 | 0.782(0.771,0.792) | 0.828(0.826,0.83) | 0.498(0.475,0.521) | 0.83(0.828,0.832) |
|  |  | top10 | 0.794(0.783,0.806) | 0.86(0.857,0.862) | 0.45(0.428,0.472) | 0.863(0.861,0.866) |
|  |  | top15 | 0.778(0.765,0.79) | 0.882(0.881,0.884) | 0.385(0.365,0.405) | 0.886(0.884,0.888) |
|  |  | top20 | 0.775(0.763,0.788) | 0.875(0.873,0.877) | 0.406(0.384,0.427) | 0.879(0.877,0.881) |
|  |  | all | 0.762(0.747,0.777) | 0.877(0.875,0.878) | 0.393(0.369,0.416) | 0.88(0.878,0.882) |

| outcomes | Model | features | PPV (95%CI) | NPV (95%CI) | F score (95%CI) | Brier score (95%CI) |
| --- | --- | --- | --- | --- | --- | --- |
| Any complications | DT | top5 | 0.028(0.027,0.029) | 0.988(0.987,0.989) | 0.054(0.051,0.056) | 0.401(0.385,0.416) |
|  |  | top10 | 0.035(0.033,0.037) | 0.987(0.986,0.988) | 0.065(0.061,0.069) | 0.244(0.236,0.252) |
|  |  | top15 | 0.03(0.027,0.032) | 0.985(0.985,0.986) | 0.056(0.051,0.06) | 0.261(0.238,0.284) |
|  |  | top20 | 0.021(0.02,0.022) | 0.986(0.984,0.987) | 0.041(0.039,0.043) | 0.571(0.535,0.606) |
|  |  | all | 0.027(0.025,0.029) | 0.987(0.986,0.988) | 0.052(0.048,0.055) | 0.379(0.345,0.414) |
|  | RF | top5 | 0.035(0.034,0.037) | 0.986(0.985,0.986) | 0.065(0.062,0.067) | 0.195(0.193,0.197) |
|  |  | top10 | 0.043(0.041,0.045) | 0.985(0.985,0.985) | 0.075(0.072,0.079) | 0.135(0.132,0.137) |
|  |  | top15 | 0.045(0.043,0.046) | 0.987(0.986,0.987) | 0.08(0.077,0.083) | 0.165(0.162,0.167) |
|  |  | top20 | 0.051(0.05,0.053) | 0.988(0.988,0.988) | 0.092(0.09,0.095) | 0.166(0.164,0.168) |
|  |  | all | 0.048(0.047,0.05) | 0.989(0.989,0.989) | 0.088(0.086,0.091) | 0.195(0.193,0.197) |
|  | GBM | top5 | 0.049(0.045,0.052) | 0.984(0.984,0.984) | 0.077(0.071,0.083) | 0.082(0.08,0.084) |
|  |  | top10 | 0.047(0.044,0.05) | 0.984(0.984,0.984) | 0.076(0.071,0.082) | 0.091(0.089,0.092) |
|  |  | top15 | 0.071(0.067,0.075) | 0.985(0.985,0.985) | 0.11(0.103,0.116) | 0.071(0.07,0.073) |
|  |  | top20 | 0.067(0.06,0.073) | 0.984(0.984,0.985) | 0.099(0.089,0.108) | 0.064(0.063,0.065) |
|  |  | all | 0.065(0.061,0.069) | 0.984(0.984,0.985) | 0.099(0.092,0.106) | 0.069(0.068,0.07) |
|  | XGBoost | top5 | 0.055(0.05,0.06) | 0.983(0.983,0.984) | 0.079(0.072,0.086) | 0.061(0.059,0.062) |
|  |  | top10 | 0.044(0.041,0.047) | 0.985(0.985,0.985) | 0.076(0.071,0.08) | 0.122(0.12,0.124) |
|  |  | top15 | 0.064(0.061,0.067) | 0.986(0.986,0.987) | 0.107(0.103,0.111) | 0.101(0.099,0.103) |
|  |  | top20 | 0.071(0.068,0.075) | 0.986(0.986,0.986) | 0.115(0.109,0.121) | 0.084(0.083,0.086) |
|  |  | all | 0.07(0.068,0.073) | 0.986(0.985,0.986) | 0.112(0.108,0.116) | 0.079(0.078,0.081) |
| Cardiac effusion/tamponade | DT | top5 | 0.01(0.01,0.011) | 0.994(0.994,0.995) | 0.02(0.019,0.021) | 0.571(0.541,0.602) |
|  |  | top10 | 0.014(0.013,0.015) | 0.993(0.993,0.994) | 0.027(0.025,0.03) | 0.262(0.229,0.295) |
|  |  | top15 | 0.015(0.014,0.017) | 0.993(0.993,0.994) | 0.029(0.027,0.032) | 0.211(0.189,0.232) |
|  |  | top20 | 0.015(0.014,0.016) | 0.993(0.993,0.994) | 0.029(0.027,0.031) | 0.211(0.189,0.232) |
|  |  | all | 0.008(0.008,0.009) | 0.992(0.991,0.992) | 0.016(0.015,0.017) | 0.409(0.379,0.439) |
|  | RF | top5 | 0.031(0.03,0.032) | 0.994(0.994,0.994) | 0.056(0.054,0.059) | 0.082(0.081,0.083) |
|  |  | top10 | 0.032(0.03,0.034) | 0.993(0.993,0.993) | 0.056(0.053,0.06) | 0.07(0.068,0.071) |
|  |  | top15 | 0.028(0.026,0.03) | 0.994(0.993,0.994) | 0.051(0.047,0.055) | 0.091(0.089,0.092) |
|  |  | top20 | 0.027(0.025,0.029) | 0.994(0.993,0.994) | 0.049(0.045,0.054) | 0.098(0.096,0.1) |
|  |  | all | 0.026(0.023,0.029) | 0.993(0.993,0.993) | 0.047(0.042,0.052) | 0.078(0.076,0.08) |
|  | GBM | top5 | 0.049(0.042,0.057) | 0.993(0.992,0.993) | 0.072(0.062,0.083) | 0.029(0.028,0.03) |
|  |  | top10 | 0.042(0.037,0.047) | 0.993(0.992,0.993) | 0.063(0.055,0.07) | 0.031(0.03,0.031) |
|  |  | top15 | 0.032(0.028,0.037) | 0.993(0.993,0.993) | 0.055(0.047,0.063) | 0.055(0.054,0.056) |
|  |  | top20 | 0.032(0.027,0.037) | 0.993(0.993,0.993) | 0.055(0.047,0.063) | 0.055(0.054,0.056) |
|  |  | all | 0.027(0.024,0.03) | 0.993(0.993,0.993) | 0.047(0.041,0.053) | 0.062(0.061,0.063) |
|  | XGBoost | top5 | 0.017(0.016,0.017) | 0.996(0.996,0.996) | 0.033(0.032,0.034) | 0.319(0.308,0.329) |
|  |  | top10 | 0.018(0.018,0.019) | 0.996(0.996,0.996) | 0.035(0.034,0.036) | 0.291(0.283,0.299) |
|  |  | top15 | 0.017(0.017,0.018) | 0.996(0.995,0.996) | 0.034(0.033,0.035) | 0.301(0.294,0.307) |
|  |  | top20 | 0.018(0.017,0.018) | 0.996(0.995,0.996) | 0.034(0.033,0.036) | 0.293(0.289,0.297) |
|  |  | all | 0.015(0.014,0.015) | 0.995(0.995,0.995) | 0.029(0.028,0.03) | 0.339(0.331,0.347) |
| Hemorrhage/hematoma | DT | top5 | 0.019(0.018,0.021) | 0.995(0.994,0.995) | 0.037(0.035,0.04) | 0.193(0.186,0.201) |
|  |  | top10 | 0.021(0.019,0.023) | 0.995(0.994,0.995) | 0.04(0.037,0.044) | 0.167(0.157,0.177) |
|  |  | top15 | 0.017(0.016,0.018) | 0.995(0.994,0.995) | 0.033(0.03,0.035) | 0.23(0.216,0.244) |
|  |  | top20 | 0.019(0.017,0.021) | 0.994(0.994,0.994) | 0.036(0.033,0.039) | 0.163(0.152,0.173) |
|  |  | all | 0.016(0.014,0.018) | 0.994(0.994,0.995) | 0.031(0.027,0.034) | 0.242(0.228,0.256) |
|  | RF | top5 | 0.036(0.034,0.039) | 0.995(0.995,0.996) | 0.068(0.064,0.072) | 0.109(0.108,0.11) |
|  |  | top10 | 0.03(0.029,0.032) | 0.995(0.994,0.995) | 0.056(0.053,0.059) | 0.111(0.109,0.113) |
|  |  | top15 | 0.038(0.037,0.04) | 0.995(0.995,0.995) | 0.071(0.067,0.075) | 0.097(0.096,0.098) |
|  |  | top20 | 0.038(0.036,0.04) | 0.995(0.995,0.995) | 0.07(0.067,0.074) | 0.093(0.091,0.094) |
|  |  | all | 0.035(0.034,0.037) | 0.994(0.994,0.995) | 0.065(0.062,0.068) | 0.086(0.085,0.088) |
|  | GBM | top5 | 0.07(0.065,0.076) | 0.993(0.993,0.993) | 0.097(0.089,0.104) | 0.023(0.022,0.024) |
|  |  | top10 | 0.069(0.062,0.076) | 0.993(0.993,0.993) | 0.098(0.088,0.108) | 0.025(0.024,0.026) |
|  |  | top15 | 0.138(0.129,0.147) | 0.993(0.993,0.993) | 0.148(0.140,0.157) | 0.015(0.014,0.015) |
|  |  | top20 | 0.13(0.123,0.138) | 0.993(0.993,0.993) | 0.138(0.132,0.145) | 0.015(0.014,0.015) |
|  |  | all | 0.12(0.111,0.13) | 0.993(0.993,0.993) | 0.129(0.12,0.139) | 0.015(0.015,0.016) |
|  | XGBoost | top5 | 0.023(0.022,0.024) | 0.995(0.995,0.995) | 0.044(0.042,0.046) | 0.172(0.17,0.174) |
|  |  | top10 | 0.026(0.025,0.027) | 0.995(0.995,0.995) | 0.049(0.047,0.051) | 0.14(0.138,0.143) |
|  |  | top15 | 0.027(0.025,0.028) | 0.994(0.994,0.995) | 0.050(0.048,0.052) | 0.118(0.116,0.119) |
|  |  | top20 | 0.026(0.025,0.028) | 0.995(0.994,0.995) | 0.050(0.047,0.052) | 0.125(0.123,0.127) |
|  |  | all | 0.026(0.024,0.027) | 0.994(0.994,0.995) | 0.049(0.046,0.051) | 0.123(0.122,0.125) |

**Figure S2: Top ranked features derived from three machine learning models**

A: Top 10 features any complication; B: Top 5 features of cardiac effusion/tamponade; C: Top 10 features of hemorrhage.

From outside to inside, the importance of feature was successively decreased.

**Figure S3 SHAP summary plot of the machine learning models for different outcomes.**

A: the top 10 features of the RF model in predicting any complication;

B: the top 5 features of the XGBoost model in predicting cardiac effusion/tamponade;

C: the top 10 features of the RF model in predicting hemorrhage.
